# Complete genome sequence analysis of *Archaeoglobus fulgidus* strain 7324 (DSM 8774), a hyperthermophilic archaeal sulfate reducer from a North Sea oil field

**DOI:** 10.1186/s40793-017-0296-5

**Published:** 2017-12-16

**Authors:** Nils-Kåre Birkeland, Peter Schönheit, Lianna Poghosyan, Anne Fiebig, Hans-Peter Klenk

**Affiliations:** 1 Department of Biology, University of Bergen, P.O. Box 7803, NO-5020 Bergen, Norway; 2 Institut für Allgemeine Mikrobiologie, Christian-Albrechts-Universität Kiel, 24118 Kiel, Germany; 3 Leibniz-Institut DSMZ – German Collection of Microorganisms and Cell Cultures, Inhoffenstrasse 7b, 38124 Braunschweig, Germany; 4 Present Address: IPK Gatersleben, Corrensstr. 3, 06466 Gatersleben, Germany; 5 Present Address: School of Biology, Newcastle University, Newcastle upon Tyne, NE1 7RU UK

**Keywords:** Anaerobic, Hyperthermophile, Sulfate reduction, SRB, Petroleum, Oil-field

## Abstract

*Archaeoglobus fulgidus* is the type species of genus *Archaeoglobus* Stetter 1998, a hyperthermophilic sulfate reducing group within the *Archaeoglobi* class of the euryarchaeota phylum. Members of this genus grow heterotrophically or chemolithoautotrophically with sulfate or thiosulfate as electron acceptors. Except for *A. fulgidus* strain 7324 and the candidate species “*Archaeoglobus lithotrophicus*”, which both originate from deep oil-fields, the other members of this genus have been recovered from marine hydrothermal systems. Here we describe the features of the *A. fulgidus* strain 7324 genome as compared to the *A. fulgidus* VC16 type strain. The 2.3 Mbp genome sequence of strain 7324 shares about 93.5% sequence identity with that of strain VC16^T^ but is about 138 Kbp longer, which is mostly due to two large ‘insertions’ carrying one extra *cdc6* (cell-cycle control protein 6) gene, extra CRISPR elements and mobile genetic elements, a high-GC ncRNA gene (*hgc*C) and a large number of hypothetical gene functions. A comparison with four other *Archaeoglobus* spp. genomes identified 1001 core *Archaeoglobus* genes and more than 2900 pan-genome orthologous genes.

## Introduction

10.1601/nm.407 strain 7324 was recovered from hot oil-field water originating from a deep oil-well in the North Sea [1]. It shares many features with the 10.1601/nm.407 type strain VC16, e.g. dissimilatory sulfate reduction, utilization of lactate and pyruvate as carbon sources, irregular coccoid to disc-shaped cells, and blue-green fluorescence under the UV microscope due to the presence of Coenzyme F_420_. Strain VC16^T^ was isolated from a shallow marine hydrothermal system at Volcano island, Italy [2]. The complete genome sequence of strain VC16^T^ was reported in 1997 as the third archaeal genome to be fully sequenced [3] and 10.1601/nm.407 has since served as a prototype for studies of archaeal and hyperthermophilic sulfate reduction [4]. Here we report a summary of the features of 10.1601/nm.407 strain 7324, together with the description of the complete genomic sequencing and annotation and comparison with the genome of the 10.1601/nm.407 type strain and other 10.1601/nm.406 spp.

## Organism information

### Classification and features

**Fig. 1:**
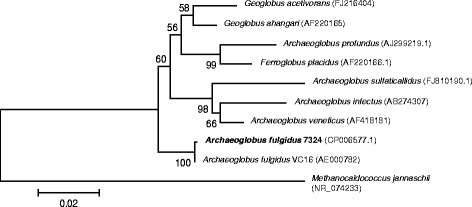
16S rRNA-based phylogenetic tree showing the position of the *Archaeoglobus fulgidus* strains within the *Archaeoglobaceae* family, using *Methanocaldococcus jannaschii* as outgroup. Only species with validly published names are included. The sequences were aligned using Clustal X [46] and the tree was inferred using the Neighbor-joining algorithm in MEGA 6.06 [47]. The bar indicated number of substitutions per site. Bootstrap values ≥60% are indicated at nodes and are based on 100 replicates. Sequence accession numbers are indicated in brackets. *A. fulgidus* strain 7324 is in bold

**Fig. 2:**
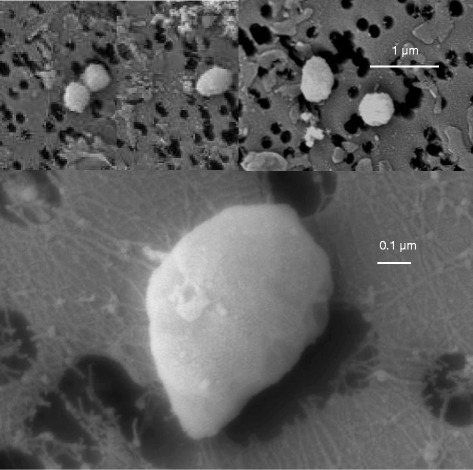
Scanning electron micrographs of cells of *A. fulgidus* strain 7324. Bars equal 1 μm (upper panel) or 0.1 μm (lower panel)

Genus 10.1601/nm.406 comprises five validly published species; 10.1601/nm.407 [2], 10.1601/nm.408 [5], 10.1601/nm.409 [6], 10.1601/nm.13216 [7], 10.1601/nm.20236 [8], and one candidate species termed “10.1601/nm.406 *lithotrophicus*” [9]. All are hyperthermophilic sulfate-reducers capable of heterotrophic or chemolitoautotrophic growth on H_2_ and CO_2_. The 10.1601/nm.406 ‘clade’ also encompasses a few non-sulfate reducing anaerobic hyperthermophiles; 10.1601/nm.14806 [10] and “10.1601/nm.413” [11, 12], which are both Fe(III) reducers, and 10.1601/nm.411, which is capable of using ferrous iron, H_2_ and sulfide as electron donors with nitrate as electron acceptor [13]. Fig. 1 shows the phylogenetic affiliation of all current members of the *Archaeoglobaceae* family, including strain 7324. All 10.1601/nm.406 species form small irregularly shaped cells. A scanning electron micrograph of 10.1601/nm.407 strain 7324 is shown in Fig. 2, revealing a similar cell shape as originally determined by transmission electron microscopy [1]. Strain 7324 has not been phylogenetically characterized by 16S rRNA gene sequencing before, but a wet lab genomic DNA: DNA hybridization with 10.1601/nm.407 strain Z, which, like the type strain, was recovered from the Vulcano island [14], revealed a genome hybridization value of 100% [1]. This close relationship was now confirmed via digital DNA-DNA hybridization [15] between strains VC16^T^ and 7324 with a GLM-based DDH estimate of 93.9%. All three 10.1601/nm.407 strains share common physiological characteristics, like growth from 60 °C to above 84 °C, use of sulfate and thiosulfate as electron acceptors, optimal growth with lactate or pyruvate as carbon sources, and production of trace amounts of methane. Although the optimal growth temperature of strain 7324 was initially determined to 76 °C, we have routinely been cultivating it at 80 °C. In contrast to the other isolates, strain 7324 rapidly lyses after the stationary phase [1]. The main features of the organism are listed in Table 1.

**Table 1:** Classification and general features of *Archaeoglobus fulgidus* strain 7324 (DSM 8774)

MIGS ID	Property	Term	Evidence code^a^
	Current classification	Domain *Archaea*	TAS [40]
		Phylum *Euryarchaeota*	TAS [41]
		Class *Archaeoglobi*	TAS [42]
		Order *Archaeoglobales*	TAS [43]
		Family *Archaeoglobaceae*	TAS [44]
		Genus *Archaeoglobus*	TAS [2]
		Species *Archaeoglobus fulgidus*	TAS [2]
		Strain 7324	TAS [1]
	Gram stain	Negative	IDA
	Cell shape	Irregular coccoid to disc shaped	TAS [1]
	Motility	Non-motile	TAS [1]
	Sporulation	Nonsporulating	NAS
	Temperature range	60 to 85 °C	TAS [1]
	Optimum temperature	76 °C^b^	TAS [1]
	Carbon source	Lactate, pyruvate, valerate	TAS [1]
	Energy source	Lactate, pyruvate, valerate + H_2_	TAS [1]
	Terminal electron receptor	Sulfate and thiosulfate	TAS [1]
MIGS-6	Habitat	Deep oil-fields	TAS [1]
MIGS-6.3	Salinity	3–500 mM NaCl (optimum at 300 mM)	TAS [1]
MIGS-22	Oxygen	Strictly anaerobic	TAS [1]
MIGS-15	Biotic relationship	Free-living	TAS [1]
MIGS-14	Pathogenicity	None	NAS
MIGS-4	Geographic location	Norway/North Sea	TAS [1]
MIGS-5	Sample collection time	1993 or earlier	TAS [1]
MIGS-4.3	Depth	Subsurface	TAS [1]

^a^Evidence codes – *IDA* Inferred from Direct Assay, *TAS* Traceable Author Statement (i.e., a direct report exists in the literature), *NAS,* Non-traceable Author Statement (i.e., not directly observed for the living, isolated sample, but based on a generally accepted property for the species, or anecdotal evidence). These evidence codes are from the Gene Ontology project [45]

^b^The strain grows well and has been routinely cultivated the last two decades in our labs at 80 °C

## Genome sequencing information

### Genome project history

10.1601/nm.407 *strain* 7324 was chosen for whole genome sequencing because it was isolated from a deep and hot oil reservoir while the closely related type strain, VC-16, was isolated from a shallow marine hot vent. A genome comparison might reveal particular adaptations of strain 7324 to the deep biosphere. The genome project information is given in the Genomes OnLine Database (Gp0102124). The genome sequence is deposited in GenBank (CP006577.1). A summary of the project information is shown in Table 2.

**Table 2:** Project information

MIGS ID	Property	Term
MIGS 31	Finishing quality	Finished
MIGS-28	Libraries used	454 Titanium paired-end, Solexa paired end
MIGS 29	Sequencing platforms	454-GS-FLX, Solexa, Illumina
MIGS 31.2	Fold coverage	103×
MIGS 30	Assemblers	Newbler, Velvet v. 1.0.18; Consed v.20.0
MIGS 32	Gene calling method	GeneMark
	Locus Tag	AFULGI
	Genbank ID	CP006577.1
	GenBank Date of Release	01.10.2014
	GOLD ID	Gp0102124
	BIOPROJECT	PRJNA208006
MIGS 13	Source Material Identifier	DSM 8774
	Project relevance	Environmental, evolution of anaerobic respiration

### Growth conditions and genomic DNA preparation

10.1601/nm.407 strain 7324 was from our own collection at the University of Bergen. It was cultivated in anaerobic medium containing lactate and sulfate as described previously [1]. The incubation temperature was 80 °C. Genomic DNA was isolated using a modification of the cetyl trimethylammonium bromide method as described [16].

### Genome sequencing and assembly

The genome was sequenced using a combination of Illumina and 454 sequencing platforms. All general aspects of library construction and sequencing can be found at the JGI website [17]. The initial assembly of 454 raw data suggested a contamination of the sequenced sample. Using blast search, all contigs (>500 nt in length) could be assigned either to 10.1601/nm.407 or 10.1601/nm.387, an archaeon that shares the same habitat [18]. To overcome this issue, two additional blast searches including all contigs longer than 500 nt were performed against the previously sequenced genome of 10.1601/nm.407 VC-16^T^ (NCBI/GenBank:AE000782) and all available genomic sequences of 10.1601/nm.374 species in Genebank (Dec. 2010). Only sequences in length sharing more than 90% sequence identity with 10.1601/nm.407 VC-16^T^ and having no hits in the 10.1601/nm.374 blast database were kept. A total of 84 Newbler contigs could be assigned to 10.1601/nm.407. Illumina raw reads were assembled to 223 contigs. Both draft assemblies were merged in a hybrid approach using the phred/phrap/consed pipeline [19]. After manual curation, a total of 27 ordered gaps were closed by bridging PCRs at LGC Genomics (Berlin). The final consensus sequence represents a single circular chromosomal element (103× coverage).

### Genome annotation

Coding genes were predicted by GeneMark [20] as part of the genome annotation pipeline in the Integrated Microbial Genomes Expert Review system [21]. The tRNAs were identified by tRNAScan-SE-1.23 [22], while ribosomal RNA genes within the genome were predicted using the tool RNAmmer [23]. Other non-coding RNA genes were predicted using Infernal [24]. CRISPR elements were identified by the program CRT [25]. Manual functional annotation was performed within the IMG platform [21] and the Artemis Genome Browser [26].

## Genome properties

**Fig. 3:**
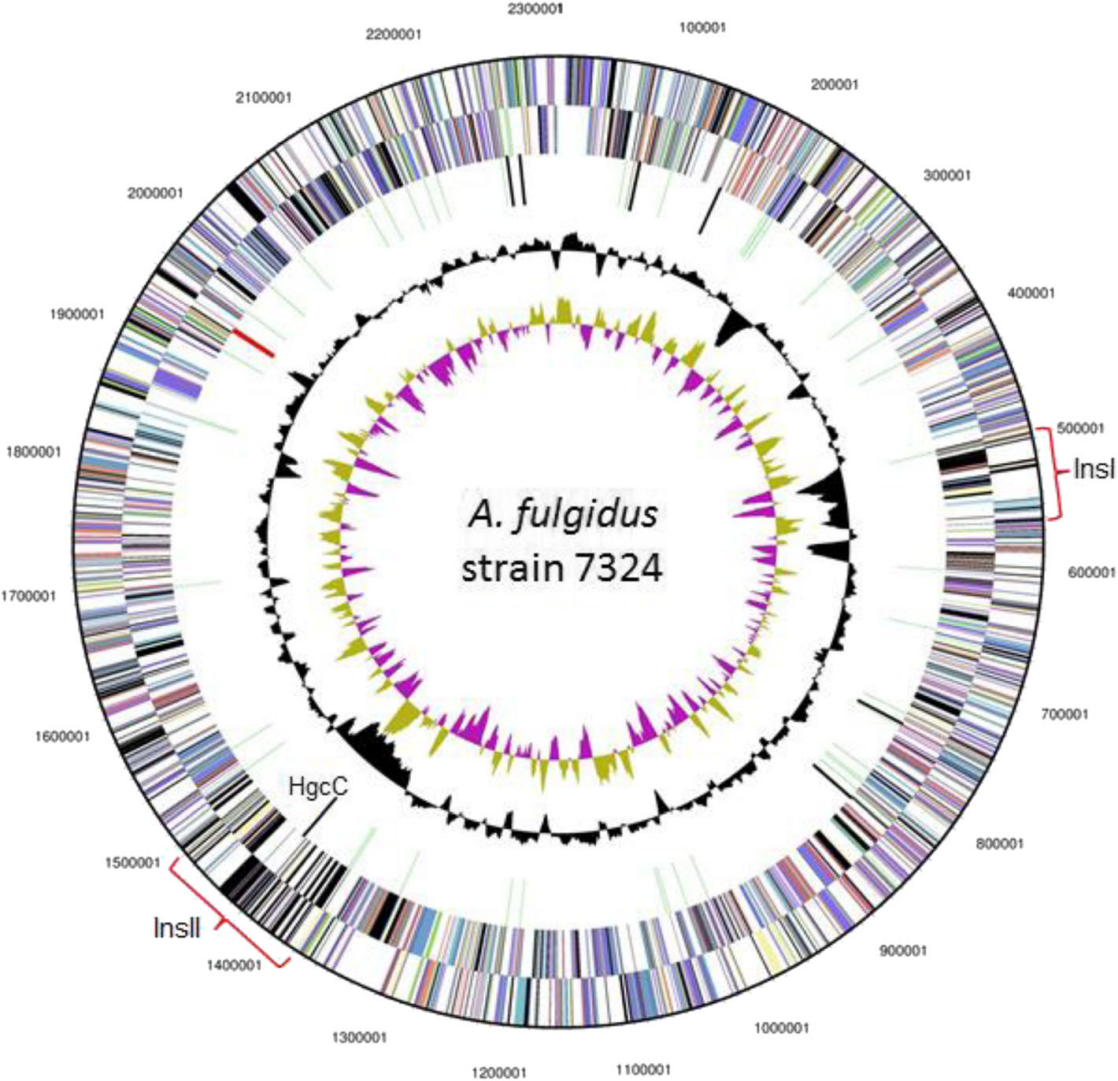
Circular map of the *A. fulgidus* strain 7324 genome. From outside to the center: Genes on forward strand (colored by COG categories), genes on the reverse strand (colored by COG categories), RNA genes (tRNAs green, rRNAs red, other RNAs black), GC content, GC skew. The non-coding RNA gene, *hgcC,* is indicated, as well as the large insertions (denoted InsI and InsII), as compared to *A. fulgidus* VC16^T^

The genome of 10.1601/nm.407 strain 7324 comprises one circular chromosome with a total size of 2,316,287 bp, which is 137,887 bp larger than 10.1601/nm.407 VC16^T^ 10.1601/strainfinder?urlappend=%3Fid%3DDSM+3404 [3]. The mole percent G + C is 48.08, which is slightly higher than the 47% value estimated previously with thermal denaturation [1] and slightly lower than for the type strain 10.1601/strainfinder?urlappend=%3Fid%3DDSM+3404 (48.6%); in any case within the 1% threshold with the species’ type strain VC16^T^ sensu Meier-Kolthoff et al. [27]. No plasmids were detected. The strain 7324 genome is the largest of the genome-sequenced 10.1601/nm.406 species, the smallest one being the 10.1601/nm.408 genome with a total size of 1.56 Mbp [28]. Out of the total 2615 genes annotated in the 7324 genome, 2558 were identified as protein coding genes and 56 as RNA genes (Table 3). Only 67.29% of the genes could be assigned to COG functional categories as listed in Table 4. Five CRISPR repeat regions were identified, as compared with only three in strain VC16^T^ (AE000782). There is only one rRNA operon (Fig. 3). As for VC16^T^, there is no apparent GC skew in the genome, which could indicate the presence of multiple DNA replication origins and explain previous difficulties in precise mapping of replication origin(s) in this species using a marker rescue analysis approach [29, 30].

**Table 3:** Genome statistics

Attribute	Value	% of Total
Genome size (bp)	2,316,287	n/a
DNA coding (bp)	2,077,792	89.70
DNA G + C (bp)	1,113,590	48.08
DNA scaffolds	1	n/a
Total genes	2615	100
Protein coding genes	2558	97.86
RNA genes	56	2.14
Pseudo genes	1	0.04
Genes in internal clusters	342	13.08
Genes with function prediction	1880	71.89
Genes assigned to COGs	1759	67.29
Genes with Pfam domains	1982	75.82
Genes with signal peptides	80	3.06
Genes with transmembrane helices	490	18.75
CRISPR repeats	5	n/a

**Table 4:** Number of genes associated with general COG functional categories

Code	Value	%age	Description
J	195	10.3	Translation, ribosomal structure and biogenesis
A	1	0.05	RNA processing and modification
K	90	4.75	Transcription
L	75	3.96	Replication, recombination and repair
B	7	0.37	Chromatin structure and dynamics
D	15	0.79	Cell cycle control, Cell division, chromosome partitioning
V	68	3.59	Defense mechanisms
T	54	2.85	Signal transduction mechanisms
M	42	2.22	Cell wall/membrane biogenesis
N	19	1	Cell motility
U	19	1	Intracellular trafficking and secretion
O	68	3.59	Posttranslational modification, protein turnover, chaperones
C	178	9.4	Energy production and conversion
G	48	2.54	Carbohydrate transport and metabolism
E	150	7.92	Amino acid transport and metabolism
F	64	3.38	Nucleotide transport and metabolism
H	138	7.29	Coenzyme transport and metabolism
I	111	5.86	Lipid transport and metabolism
P	82	4.33	Inorganic ion transport and metabolism
Q	32	1.69	Secondary metabolites biosynthesis, transport and catabolism
R	249	13.15	General function prediction only
S	170	8.98	Function unknown
–	855	32.71	Not in COGs

## Insights from the genome sequence

**Fig. 4:**
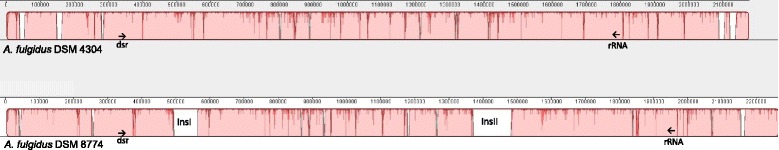
MAUVE version 20,150,226 [48] alignment of the *A. fulgidus* strains VC-16^T^ (upper) and 7324 (lower) chromosomes. The large insertions in the 7324 chromosomes are indicated as regions InsI and InsII. Approximate positions of the rRNA and *dsr* (dissimilatory sulfite reductase) genes are indicated by arrows

**Fig. 5:**
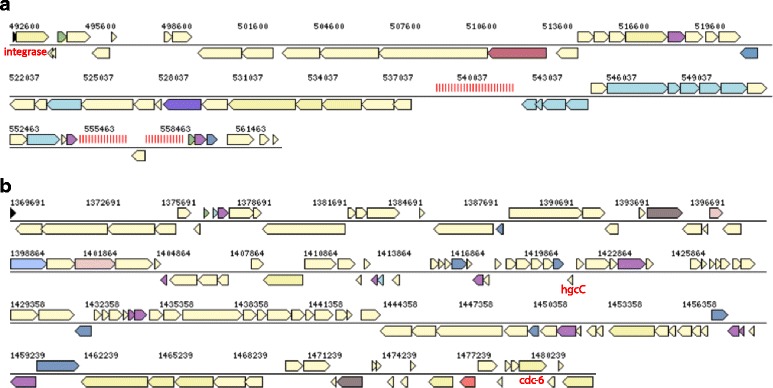
Genetic structure of the large insertions in strain 7324; **a** InsI; **b** InsII. Genes are colored according to COG color codes

Genes encoding central metabolic pathways like dissimilatory sulfate reduction, lactate oxidation, a complete TCA cycle and the Acetyl-CoA pathway were identified in strain 7324, confirming previous physiological characterization and similarity with strain VC16^T^ [1]. A genome alignment between strains VC16^T^ and 7324 revealed a large degree of genomic similarity and sequence synteny, interrupted mostly by two large additional regions of about 64 and 109 Kbp (InsI and InsII, respectively) in strain 7324 as compared to VC16^T^ (Fig. 4). Both these regions are flanked by a disrupted tRNA gene, which implies that InsI and II represent genomic insertions. They also possess a considerably lower G + C content (42%) as compared to the average of the genome (Fig. 3), indicating a result of recombination with an AT-rich organism. This is further supported by identification of a non-coding high-GC RNA gene in InsII belonging to the *hgcC* family of ncRNA (RFAM v12 accession code RF00062) typically found in AT-rich hyperthermophiles (Figs. 3 and 5b). This ncRNA family was originally identified in the genomes of 10.1601/nm.399 and 10.1601/nm.168 [31] but its function is still unresolved.

InsII also contains a gene encoding an extra homologue of the Orc1/Cdc6 family of replication initiation control proteins in addition to the two other *cdc6* homologous in the 7324 and VC16^T^ genomes. The closest homologue identified by a blast search is from 10.1601/nm.409 (62% amino acid sequence identity). The majority of the other genes are hypothetical or have a general function prediction only. InsI carries two CRISPR repeat regions and 14 genes encoding CRISPR-associated proteins including a Cas6 homologue (Fig. 5a). The rest of this insert mostly contains hypothetical genes.

**Fig. 6:**
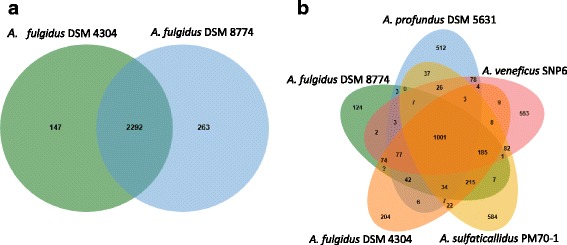
Venn diagrams showing the distribution of orthologous and unique genes for **a** *A. fulgidus* strains DSM 4304 and DSM 8774, and **b** for all the completely genome sequenced *Archaeoglobus* representatives; *A. fulgidus* DSM 4305, *A. fulgidus* DSM 8774, Archaeoglobus profundus DSM 5631, *Archaeoglobus veneficus* DSM 11195 and Archaeoglobus sulfaticallidus DSM 19444. The diagrams were prepared using ‘jvenn’ [49] as implemented in the EzBioCloud’s Comparative Genomics Database [50]

A Venn diagram shows that 10.1601/nm.407 strains VC16^T^ and 7324 share a large number of genes (2292) (Fig. 6a), reflecting the high degree of genome similarity. The 263 genes unique to strain 7324 include about 200 hypothetical genes/uncharacterized functions most of them belonging to the large insertions and the CRISPR-associated genes of InsII. The Venn diagram including all the five genome-sequenced strains revealed an 10.1601/nm.406 core genome of 1001 genes (Fig. 6b), most of which encode energy-yielding, biosynthetic and regulatory functions. About 200 of the core genes belong to the hypothetical/uncharacterized category in the EggNog database [32]. This is considerably lower than the 32% fraction of unassigned genes for the entire strain 7324, but underpins that a large part of central gene functions in this genus still remain to be disclosed. About 2900 genes belong to the 10.1601/nm.406 pan-genome, being unique to one of the genomes or shared by 2 to 4 of the species.

10.1601/nm.407 strain 7324 has been reported to degrade starch [33] and several enzymes involved in starch degradation have been purified from cells grown on starch. These enzymes include cyclodextrin gluconotransferase, cyclodextrinase, maltodextrin phosphorylase, and phosphoglucomutase, ADP-dependent glucokinase, ADP-dependent phosphofructokinase and pyruvate kinase [34–37]. The enzymes were biochemically characterized and the N-termini (with exception of cyclodextrinase) have been determined. They showed highest sequence identity to proteins from 10.1601/nm.372, e.g. from 10.1601/nm.387 *.* However, in the genome of 10.1601/nm.407 presented here, genes encoding sugar degrading enzymes have not been identified. Rather, various analyses indicated that 10.1601/nm.407 strain 7324, deposited as 10.1601/strainfinder?urlappend=%3Fid%3DDSM+8774, was contaminated with a 10.1601/nm.374 strain. The genome of this 10.1601/nm.374 strain has also been sequenced and all genes encoding the sugar degrading enzymes, originally attributed to 10.1601/nm.407 strain 7324, were found in the 10.1601/nm.374 strain genome. Also, glutamate dehydrogenase from a 10.1601/strainfinder?urlappend=%3Fid%3DDSM+8774 culture grown in the presence of small amounts of yeast extract (0.3 gL^−^) was previously purified and characterized [38, 39], but appears also to be encoded by the 10.1601/nm.374 strain genome. The purity of the original 10.1601/nm.407 7324 isolate was not assessed by 16S rRNA gene sequencing prior to deposition at DSMZ [1] and whether the 10.1601/nm.374 contamination was present in the original culture or has been introduced at a different stage is not known. The genome analysis of this 10.1601/nm.374 strain, which appears to represent a novel 10.1601/nm.374 species, will be published separately.

## Conclusions

The complete genome of 10.1601/nm.407 strain 7324, recovered from hot water produced from an oil well in the North Sea was sequenced and annotated. In addition to the 10.1601/nm.407 type strain, VC16, isolated from a shallow hot vent in the Mediterranean, this is the second 10.1601/nm.407 genome to be characterized. The two strains share 93.5% genome sequence similarity, and differ mostly by two large insertions of 64 and 109 Kbp in strain 7324 that seem to have originated from an AT-rich archaeon. The insertions carry two additional CRISPR elements, an extra *cdc6* gene, a variety of mobile genetic elements and a large number of hypothetical and unassigned genes. Based on comparison with four other 10.1601/nm.406 spp. genomes, the 10.1601/nm.406 core genome was estimated to 1001 genes. No particular traits indicating adaptation to the petroleum reservoir subsurface environment could be identified.
